# Degradation of Photoreceptor Outer Segments by the Retinal Pigment Epithelium Requires Pigment Epithelium-Derived Factor Receptor (PEDF-R)

**DOI:** 10.1167/iovs.62.2.30

**Published:** 2021-02-19

**Authors:** Jeanee Bullock, Federica Polato, Mones Abu-Asab, Alexandra Bernardo-Colón, Elma Aflaki, Martin-Paul Agbaga, S. Patricia Becerra

**Affiliations:** 1 Section of Protein Structure and Function, Laboratory of Retinal Cell and Molecular Biology, National Eye Institute, National Institutes of Health, Bethesda, Maryland, United States; 2 Department of Biochemistry and Molecular & Cellular Biology, Georgetown University Medical Center, Washington DC, United States; 3 Section of Histopathology, National Eye Institute, National Institutes of Health, Bethesda, Maryland, United States; 4 Departments of Cell Biology and Ophthalmology, Dean McGee Eye Institute, University of Oklahoma Health Sciences Center, Oklahoma City, Oklahoma, United States

**Keywords:** PNPLA2, RPE, phagocytosis, outer segments, PEDF-R

## Abstract

**Purpose:**

To examine the contribution of pigment epithelium-derived factor receptor (PEDF-R) to the phagocytosis process. Previously, we identified PEDF-R, the protein encoded by the *PNPLA2* gene, as a phospholipase A2 in the retinal pigment epithelium (RPE). During phagocytosis, RPE cells ingest abundant phospholipids and protein in the form of photoreceptor outer segment (POS) tips, which are then hydrolyzed. The role of PEDF-R in RPE phagocytosis is not known.

**Methods:**

Mice in which *PNPLA2* was conditionally knocked out (cKO) in the RPE were generated. Mouse RPE/choroid explants were cultured. Human ARPE-19 cells were transfected with si*PNPLA2* silencing duplexes. POSs were isolated from bovine retinas. The phospholipase A2 inhibitor bromoenol lactone was used. Transmission electron microscopy, immunofluorescence, lipid labeling, pulse–chase experiments, western blots, and free fatty acid and β-hydroxybutyrate assays were performed.

**Results:**

The RPE of the cKO mice accumulated lipids, as well as more abundant and larger rhodopsin particles, compared to littermate controls. Upon POS exposure, RPE explants from cKO mice released less β-hydroxybutyrate compared to controls. After POS ingestion during phagocytosis, rhodopsin degradation was stalled both in cells treated with bromoenol lactone and in *PNPLA2-*knocked-down cells relative to their corresponding controls. Phospholipase A2 inhibition lowered β-hydroxybutyrate release from phagocytic RPE cells. *PNPLA2* knockdown also resulted in a decline in fatty acids and β-hydroxybutyrate release from phagocytic RPE cells.

**Conclusions:**

PEDF-R downregulation delayed POS digestion during phagocytosis. The findings imply that the efficiency of RPE phagocytosis depends on PEDF-R, thus identifying a novel contribution of this protein to POS degradation in the RPE.

A vital function of the retinal pigment epithelium (RPE) is to phagocytose the tips of the photoreceptors in the neural retina. As one of the most active phagocytes in the body, RPE cells ingest daily a large amount of lipids and protein in the form of photoreceptor outer segment (POS) tips.^1^^–^^5^ As outer segments are constantly being renewed at the base of photoreceptors, the ingestion of POS tips (∼10% of an outer segment) by RPE cells serves to balance outer segment renewal, which is necessary for the visual activity of photoreceptors. The ingested POSs serve as an abundant source of fatty acids, which are substrates for fatty acid β-oxidation and ketogenesis to support the energy demands of the RPE.^6^^–^^8^ The fatty acids liberated from phagocytosed POSs are also used as essential precursors for lipid and membrane synthesis and as bioactive mediators in cell signaling processes; for example, the main fatty acid in POS phospholipids is docosahexaenoic acid, which is involved in signaling in the retina.^9^ Rhodopsin, a pigment present in rod photoreceptors involved in visual phototransduction, is the most abundant protein in POS. Approximately 85% of the total protein of isolated bovine POS is rhodopsin,^10^ which is embedded in a phospholipid bilayer at a molar ratio between rhodopsin and phospholipids of about 1:60.^11^ Conversely, the RPE lacks expression of the rhodopsin gene. The importance of POS clearance by the RPE in the maintenance of photoreceptors was demonstrated in an animal model for retinal degeneration, the Royal College Surgeons (RCS) rats, in which a genetic defect in the RCS rats renders their RPE unable to effectively phagocytose POS, thereby leading to rapid photoreceptor degeneration.^12^^,^^13^ Moreover, human RPE phagocytosis declines moderately with age, and the decline is significant in RPE of human donors with age-related macular degeneration, underscoring its importance in this disease.^14^ Therefore, there is increasing interest in studying regulatory hydrolyzing enzymes involved in RPE phagocytosis for maintaining retina function and the visual process.

We have previously reported that the human RPE expresses the *PNPLA2* gene, which encodes a 503-amino acid polypeptide that exhibits phospholipase A2 (PLA2) activity and is referred to as pigment epithelium-derived factor receptor (PEDF-R).^15^ The enzyme liberates fatty acids from phospholipids, specifically those in which docosahexaenoic acid (DHA) is in the *sn*-2 position.^16^ RPE plasma membranes contain the PEDF-R protein,^15^^,^^17^ and photoreceptor membrane phospholipids have a high content of DHA in their *sn*-2 position,^9^ suggesting that upon POS ingestion the substrate lipid is available to interact with PEDF-R. Other laboratories have used different names for the PEDF-R protein (e.g., iPLA2ζ, desnutrin, adipose triglyceride lipase), and have shown that it exhibits additional lipase activities: triglyceride lipase and acylglycerol transacylase enzymatic activities.^18^^–^^20^ In macrophages, the triglyceride hydrolytic activity is critical for efficient efferocytosis of bacteria and yeast.^21^ Interestingly, we and others have shown that the inhibitor of calcium-independent phospholipases A2 (iPLA2s), bromoenol lactone (BEL), inhibits the phospholipase and triolein lipase activities of PEDF-R/iPLA2ζ.^15^,^18^ In addition, BEL can impair the phagocytosis of POSs by ARPE-19 cells, associating phospholipase A2 activity with the regulation of photoreceptor cell renewal.^22^ However, the responsible phospholipase enzyme involved in RPE phagocytosis is not yet known.

Given that the role of PEDF-R in RPE phagocytosis has not yet been studied, here we explored its contribution in this process. We hypothesized that PEDF-R is involved in the degradation of phospholipid-rich POS in RPE phagocytosis. To test this hypothesis, we silenced the *PNPLA2* gene in vivo and in vitro. Results show that with downregulation of *PNPLA2* expression and inhibition of the PLA2 activity of PEDF-R, RPE cells can neither break down rhodopsin nor release β-hydroxybutyrate (β-HB) and fatty acids, thus identifying a novel contribution of this protein in POS degradation. We discuss the role that PEDF-R may play in the disposal of lipids from ingested OS and, in turn, in the regulation of photoreceptor cell renewal.

## Methods

### Animals

The generation of desnutrin floxed mice (hereafter referred to as *Pnpla*2^f/f^)^23^ and the Tg(*BEST1-cre*)^Jdun^ transgenic line^24^ (which we refer to as *BEST1-cre* here) have been previously reported. The desnutrin floxed transgenic mouse model was kindly donated to our laboratory by Hei Sook Sul, PhD. The transgenic Tg(*BEST1-cre*)^Jdun^ mouse model was a generous gift by Joshua Dunaief, MD, PhD. It is an RPE-specific, *cre*-expressing transgenic mouse line in which the activity of the human *BEST1* promoter is restricted to the RPE and drives the RPE-specific expression of the targeted *cre* in the eye of transgenic mice.^24^ Homozygous floxed *Pnpla*2 (*Pnpla*2^f/f^) mice were crossed with transgenic *BEST1-cre* mice. The resulting mice carrying one floxed allele and the *cre* transgene (*Pnpla*2^f/+/Cre^) were crossed with *Pnpla*2^f/f^ mice to generate mice with *Pnpla*2 knockout specifically in the RPE; these mice are homozygous floxed mice expressing the *cre* transgene only in the RPE (*Pnpla*2^f/f/Cre^; here also referred to as cKO). *Pnpla*2^f/f/Cre^ or *Pnpla*2^f/+/Cre^ were also used for breeding with *Pnpla*2^f/f^ to expand the colony. *Pnpla*2^f/+^ or *Pnpla*2^f/f^ littermates, obtained through this breeding, were used as control mice. All procedures involving mice were conducted following protocols approved by the National Eye Institute Animal Care and Use Committee and in accordance with the ARVO Statement for the Use of Animals in Ophthalmic and Vision Research. The mice were housed in the National Eye Institute animal facility with lighting at around 280 to 300 lux in cycles of 12 hours (6 AM–6 PM) light and 12 hours dark (6 PM–6 AM).

### DNA Isolation

DNA was isolated from mouse eyecups using the salt–chloroform DNA extraction method^25^ and dissolved in 200 µL of Tris-EDTA (TE), composed of 10-mM Tris-HCl (pH 8) and 1-mM EDTA. Aliquots (2 µL) of the DNA solution were then used for each PCR reaction using oligonucleotide primers P1 and P2 (sequences kindly provided by the laboratory of Hei Sook Sul PhD) (Table 1).

**Table 1.** Primers Used for qRT-PCR

Gene (Human)	Forward Primer	Reverse Primer
*PNPLA2*	5′-AGCTCATCCAGGCCAATGTCT-3′	5′-TGTCTGAAATGCCACCATCCA-3′
*18S*	5′-GGTTGATCCTGCCAGTAG-3′	5′-GCGACCAAAGGAACCATAAC-3′
*P1* and *P2*	5′-GCTTCAAACAGCTTCCTCATG-3′	5′-GGACTTTCGGTCATAGTTCCG-3′

### RNA Extraction, cDNA Synthesis, and Quantitative RT-PCR

RNA was isolated from the mouse RPE following the methodology previously described.^26^ Total RNA was purified from ARPE-19 cells using the RNeasy Mini Kit (QIAGEN Sciences, Inc., Germantown, MD, USA) following the manufacturer's instructions. Between 100 and 500 ng of total RNA was used for reverse transcription using the Invitrogen SuperScript III First-Strand Synthesis system (Thermo Fisher Scientific, Waltham, MA, USA). The *PNPLA*2 transcript levels in ARPE-19 cells determined by quantitative RT-PCR were normalized using the QIAGEN QuantiTect SYBR Green PCR Kit in the QuantStudio 7 Flex Real-Time PCR System (Thermo Fisher Scientific). The primer sequences used in this study are listed in Table 1. Murine *PNPLA*2 mRNA levels relative to *HPRT* transcript levels were measured using the QuantStudio 7 Flex Real-Time PCR System using TaqMan gene expression assays (*PNPLA*2, Mm00503040_m1; *HPRT*, Mm00446968_m1; Thermo Fisher Scientific). *PNPLA*2 relative expression to *HPRT* was calculated using the comparative ΔΔCT method.^27^

### Eyecup Flatmounts

Eyecup (RPE, choroid, sclera) flatmounts were prepared and processed as follows. After enucleation and removal of cornea, lens, and retina, eyecups were fixed for 1 hours in 4% paraformaldehyde at room temperature and washed three times for 10 minutes each in Tris-buffered saline (TBS; 25-mM Tris-HCl, pH 7.4; 137-mM NaCl; 2.7-mM KCl). They were then blocked for 1 hour with 10% normal goat serum (NGS) in 0.1% TBS-T^a^ (TBS containing 0.1% Triton-X; Sigma-Aldrich, St. Louis, MO, USA). Primary antibodies against Cre recombinase and rhodopsin (see Table 2) in 0.1% TBS-T^a^ containing 2% NGS were diluted and used at 4°C for 16 hours. Then, the eyecups were washed three times for 10 minutes each with TBS-T^a^ followed by incubation at room temperature for 1 hour with the respective secondary antibodies, using 4′,6-diamidino-2-phenylindole to counterstain the nuclei and Alexa Fluor 647 Phalloidin (Thermo Fisher Scientific) to label the RPE cytoskeleton, diluted in 0.1% TBS-T^a^ containing 2% NGS. Eyecups were then flattened by introducing incisions and mounted with Prolong Gold antifade reagent (Thermo Fisher Scientific). Images of the entire flatmounts were collected using the tiling feature of the epifluorescent Axio Imager Z1 microscope (Carl Zeiss Microscopy, White Plains, NY, USA) at 20× magnification. The collected images were stitched together using the corresponding feature of the Zen Blue software (Carl Zeiss Microscopy). Eyecups were also imaged using confocal microscopy (Zeiss LSM 700) at 20× magnification collecting *z*-stacks spanning 2 µm from each other and covering from the basal to the apical surface of the RPE cells. The image resulting from the maximum intensity projection of the *z*-stacks was employed for analysis.

**Table 2.** Antibodies Used in the Study

Antibody	Type and Host	Application	Dilution	Company	Catalog No.
GAPDH	Monoclonal mouse	WB	1:10,000	GeneTex	GTX627408
PEDF-R	Polyclonal rabbit	WB	1:1000	ProteinTech Group	55190-1-AP
		IF	1:250		
Rhodopsin (A531)	Monoclonal mouse	WB	1:5000	Novus Biologicals	NBP2-25159
		IF	1:800		
Rhodopsin (B630)	Monoclonal mouse	IF	1:1000	Novus Biologicals	NBP2-25160
Cre recombinase	Monoclonal rabbit	IF	1:800	Cell Signaling Technology	15036
Alexa Fluor 488	Goat anti-Mouse IgG (H+L)	IF	1:500	Thermo Fisher Scientific	A-11001
Alexa Fluor 555	Goat anti-Rabbit IgG (H+L)	IF	1:500	Thermo Fisher Scientific	A-21428
Alexa Fluor 647 Phalloidin	—	IF	1:100	Thermo Fisher Scientific	8940

Five regions of interest (ROIs; 520 µm × 520 µm) were selected for each image of the flatmount from cKO mice and control mice. The percentage of Cre-positive cells was determined by dividing the number of cells containing Cre-stained nuclei by the number of RPE cells in each ROI (identified by F-actin staining).

For phagocytosis assay, at least six ROIs (320.5 µm × 320.5 µm) were analyzed per mouse. Rhodopsin-stained particles were counted using ImageJ (National Institutes of Health, Bethesda, MD, USA), after adjusting the color threshold and size of the particles to eliminate the background.

### Transmission Electron Microscopy

Mouse eyes were enucleated and doubly fixed in 2.5% glutaraldehyde in PBS and 0.5% osmium tetroxide in PBS and embedded in epoxy resin. Thin sections (90 nm in thickness) were generated and placed on 200-mesh copper grids, dried for 24 hours, and double-stained with uranyl acetate and lead citrate. Sections were viewed and photographed with a JEOL JEM 1010 transmission electron microscope (JEOL, Ltd., Peabody, MA, USA).

### Electroretinography

In dim red light, overnight dark-adapted mice were anesthetized by intraperitoneal (IP) injection of ketamine (92.5 mg/kg) and xylazine (5.5 mg/kg). Pupils were dilated with a mixture of 1% tropicamide and 0.5 % phenylephrine. A topical anesthetic, tetracaine (0.5%), was administered before positioning the electrodes on the cornea for recording. ERGs were recorded from both eyes by the Espion E2 system with ColorDome (Diagnosys LLC, Lowell, MA, USA). Dark-adapted responses were elicited with increasing light impulses with intensity from 0.0001 to 10 candela-seconds per meter squared (cd·s/m^2^). Light-adapted responses were recorded after a 2-minute adaptation to a rod-saturating background (20 cd·s/m^2^) with light stimulus intensity from 0.3 to 100 cd·s/m^2^. During the recording, the mouse body temperature was maintained at 37°C by placing the mouse on a heating pad. The a-wave amplitudes were measured from baseline to negative peak, and the b-wave amplitudes were measured from the a-wave trough to the b-wave peak.

### Direct-Coupled ERG

For direct-coupled (DC) ERG, sliver chloride electrodes connected to glass capillary tubes filled with Hank's buffered salt solution (HBSS) were used for recording. The electrodes were kept in contact with the cornea for a minimum of 10 minutes until the electrical activity reached steady state. Responses to 7-minute steady light stimulation were recorded.

### Cell Culture

Human ARPE-19 cells (CRL-2302; American Type Culture Collection, Manassas, VA, USA) were maintained in Dulbecco's Modified Eagle Medium/Nutrient Mixture F-12 (DMEM/F-12; Thermo Fisher Scientific) supplemented in 10% fetal bovine serum (FBS) and 1% penicillin/streptomycin at 37°C with 5% CO_2_. For the assays described below, a total of 1 × 10^5^ cells in 0.5 mL were plated per well of 24-well plates and incubated for 3 days in DMEM/F-12 with 10% FBS and 1% penicillin/streptomycin. ARPE-19 cells were authenticated by Bio-Synthesis, Inc. (Lewisville, TX, USA) at passage 27. ARPE-19 cells in passage numbers 27 to 32 were used for all experiments.

### Silencing of *PNPLA2* in ARPE-19 Cells Using siRNA

Small interfering RNA (siRNA) oligo duplexes of 27 bases in length for human *PNPLA2* were purchased from OriGene Technologies, Inc. (Rockville, MD, USA). Their sequences and that of a scramble siRNA (Scr) (SR324651 and SR311349) are given in Table 3. From the six duplexes, siRNAs C, D, and E consistently provided the highest silencing efficiency; therefore, these three duplexes were used individually for silencing experiments and are referred to as si*PNPLA2*. ARPE-19 cells were transfected by reverse transfection in 24-well tissue culture plates as follows: A total of 6 pmol of siRNA was diluted in 100 µL of Gibco OptiMem (Thermo Fisher Scientific) per well and mixed with 1 µL of Invitrogen Lipofectamine RNAiMAX; mock transfected cells received only 1 µl of Lipofectamine. The mixture was then added to each well. After incubation at room temperature for 10 minutes, a total of 1 × 10^5^ cells in 500-µL antibiotic-free DMEM/F-12 containing 10% FBS was added to each well, and the plate was swirled gently to mix. Assays were performed 72 hours after transfection.

**Table 3.** siRNA Duplex Sequences

siRNA Duplex	Identifier	Duplex Sequence
SR311349A	A	rCrGrCrCrArArArGrCrArCrArUrGrUrArArUrArArArUrGCT
SR311349B	B	rGrGrCrArCrArUrArUrArGrArArCrGrUrArCrUrGrCrArUrUCC
SR311349C	C	rGrCrCrUrGrArGrArCrGrCrCrUrCrCrArUrUrArCrCrArCTG
SR324651A	D	rCrCrArArGrUrUrCrArUrUrGrArGrGrUrArUrCrUrArArAGA
SR324651B	E	rCrUrGrCrCrArCrUrCrUrArUrGrArGrCrUrUrArArGrArACA
SR324651C	F	rCrUrUrGrGrUrArArArUrArArArArArCrGrArArArArUrGTT

### Phagocytosis of Bovine POSs by ARPE-19 Cells

POSs were isolated as previously described^28^ from freshly obtained cow eyes (J.W. Treuth & Sons, Catonsville, MD, USA). POS pellets were stored at –80°C until use. Quantification of POS units was performed using trypan blue and resulted in an average of 5 × 10^7^ POS units per bovine eye. The concentration of protein from purified POSs was 21 pg/POS unit. Proteins in the POS samples resolved by sodium dodecyl sulfate–polyacrylamide gel electrophoresis (SDS-PAGE) had the expected migration pattern for both reduced and non-reduced conditions, and the main bands stained with Coomassie blue co-migrated with rhodopsin–immunoreactive proteins in western blots of POS proteins (Supplementary Fig. S1). The percentage of rhodopsin in the protein content of POS was estimated from the gels and revealed that 80% or more of the protein content corresponded to rhodopsin.

Using electrospray ionization–tandem mass spectrometry (ESI-MSMS) as previously described,^29^ we determined the lipid composition of the POSs that were fed to the ARPE-19 cells. Phagocytosis assays in ARPE-19 cells were performed as follows: ARPE-19 cells (1 × 10^5^ cells per well) were attached to 24-well plates (commercial tissue culture-treated polystyrene plates^30^ purchased from Corning, Corning, NY, USA) and cultured for 3 days to form confluent and polarized cell monolayers, as we reported previously.^31^ Ringer's solution was prepared and composed of the following: 120.6-mM NaCl, 14.3-mM NaHCO_3_, 4.2-mM KCl, 0.3-mM MgCl_2_, and 1.1-mM CaCl_2_, with 15-mM HEPES dissolved separately and adjusted to pH 7.4 with *N*-methyl-d-glucamine. Prior to use, l-carnitine was added to the Ringer's solution to achieve a 1-mM final concentration of l-carnitine. Purified POSs were diluted to a concentration of 1 × 10^7^ POSs/mL in Ringer's solution containing freshly prepared 5-mM glucose. A total of 500 µL of this solution (medium) was added to each well, and the cultures were incubated for 30 minutes, 60 minutes, or 2.5 hours at 37°C. For pulse–chase experiments, after 2.5 hours of incubation with POSs (pulse), media with POSs were removed from the wells and replaced with DMEM/F-12 containing 10% FBS, and incubation continued for a total of 16 hours. The media were separated from the attached cells and stored frozen until use; the cells were used for preparing protein extracts, and they were either used immediately or stored frozen until use. For experiments using bromoenol lactone (BEL; Sigma-Aldrich), BEL dissolved in vehicle dimethyl sulfoxide (DMSO) was mixed with Ringer's solution, and the mixture added to the cells and incubated for 1 hour prior to starting the phagocytosis assays. The mixture was removed and replaced with the POS mixture, as described above, containing DMSO or BEL during the pulse. The assays were performed in duplicate wells per condition, and each set of experiments was repeated at least two times.

### Cell Viability by Crystal Violet Staining

ARPE-19 cells were seeded in a 96-well plate at a density of 2 × 10^4^ cells per well. The cells were incubated at 37°C for 3 days. The medium was removed and replaced with Ringer's solution containing various concentrations of BEL, and incubation continued at 37°C for 3.5 hours. The medium was replaced with complete medium, and the cultures were incubated for a total of 16 hours. After two washes of the cells with deionized H_2_O, the plate was inverted and tapped gently to remove excess liquid. A total of 50 µL of a 0.1% crystal violet (Sigma-Aldrich) staining solution in 25% methanol was added to each well and incubated at room temperature for 30 minutes on a bench rocker with a frequency of 20 oscillations per minute. The cells in the wells were briefly washed with deionized H_2_O, and the plates were then inverted and placed on a paper towel to air dry without a lid for 10 minutes. For crystal violet extraction, 200 µL of methanol was added to each well, and the plate was covered with a lid and incubated at room temperature for 20 minutes on a bench rocker set at 20 oscillations per minute. The absorbance of the plate was measured at 570 nm.

### Western Blot

ARPE-19 cells plated in multiwell cell culture dishes were washed twice with ice-cold Dulbecco's phosphate-buffered saline (137-mM NaCl, 8-mM Na_2_HPO_4_-7H_2_O, 1.47-mM KH_2_PO_4_, 2.6-mM KCl, 490-µM MgCl_2_-6H_2_O, 900-µM CaCl_2_, pH 7.2). A total of 120 µL of cold radioimmunoprecipitation assay lysis and extraction buffer (Thermo Fisher Scientific) with protease inhibitors (Roche Diagnostics, Indianapolis, IN, USA), added as per manufacturer's instructions, was added to each well, and the plate was incubated on ice for 10 minutes. Cell lysates were collected and sonicated for 20 seconds with a 50% pulse (Sonic Dismembrator Model 100; Thermo Fisher Scientific), and the cellular debris was removed from soluble cell lysates by centrifugation at 20,800*g* at 4°C for 10 minutes. Protein concentration in the lysates was determined using the Pierce BCA Protein Assay Kit (Thermo Fisher Scientific), and the cell lysates were stored at –20°C until use. Between 5 and 10 µg of cell lysates were used for western blots.

Proteins were resolved by SDS-PAGE and transferred to nitrocellulose membranes for immunodetection. The antibodies used are listed in Table 2. For PEDF-R immunodetection, membranes were incubated in 1% BSA (Sigma-Aldrich) in TBS-T^b^ (50-mM Tris, pH 7.5, and 150-mM NaCl containing 0.1% Tween-20; Sigma-Aldrich) at room temperature for 1 hour. They were then incubated in a solution of primary antibody against human PEDF-R at 1:1000 in 1% BSA/TBS-T^b^ at 4°C for over 16 hours. Membranes were washed vigorously with TBS-T^b^ for 30 minutes and incubated with anti-Rabbit HRP (Kindle Biosciences, Greenwich, CT, USA) diluted 1:1000 in 1% BSA/TBS-T^b^ at room temperature for 30 minutes. The membranes were washed vigorously with TBS-T^b^ for 30 minutes, and immunoreactive proteins were visualized using the KwikQuant imaging system (Kindle Biosciences). For rhodopsin immunodetection, membranes were incubated in 5% dry milk (Nestlé USA, Arlington, VA, USA) in PBS-T (137-mM NaCl; 2.7-mM KCl; 10-mM Na_2_HPO_4_; 2-mM KH_2_PO_4_, pH 7.4; 0.1% Tween 20) at room temperature for 1 hour. The membranes were then incubated in a solution of primary antibody against human rhodopsin (Novus Biologicals LLC, Littleton, CO, USA) at 1:5000 in a suspension of 5% dry milk in PBS-T at 4°C for over 16 hours. The membranes were washed vigorously with PBS-T for 30 minutes, followed by incubation in a solution of anti-Mouse HRP (Kindle Biosciences) 1:1000 in 5% milk in PBS-T at room temperature for 30 minutes. The membranes were washed vigorously with PBS-T for 30 minutes, and immunoreactive proteins were visualized using the KwikQuant imaging system. For protein loading control, the antibodies in membranes, processed as described above, were removed using Restore Western Blot Stripping Buffer (Thermo Fisher Scientific), followed by incubation with 1% BSA in TBS-T (blocking solution) at room temperature for 1 hour and then by a solution of primary antibody against glyceraldehyde 3-phosphate dehydrogenase (GAPDH, GTX627408; GeneTex, Inc., Irvine, CA, USA) 1:10,000 in 1% BSA/TBS-T at 4°C for over 16 hours. After the membranes were washed vigorously with TBS-T at room temperature for 30 minutes, they were incubated in a solution of anti-Mouse HRP at 1:1000 in 1% BSA/TBS-T at room temperature for 30 minutes. After washes with TBS-T as described above, the immunoreactive proteins were visualized using the KwikQuant imaging system.

### β-Hydroxybutyrate Quantification Assay

In mice, the assay was performed as described before.^8^ Briefly, after removal of the cornea, lens and retina, optic nerve, and extra fat and muscles, the eyecup explant from one eye was placed in a well of a 96-well plate containing 170 µL Ringer's solution, and the eyecup from the contralateral eye was placed in another well with the same volume of Ringer's solution containing 5-mM glucose and purified bovine POSs (200-µM phospholipid content, a kind gift from Dr. Kathleen Boesze-Battaglia, PhD). The eyecup explant cultures were then incubated for 2 hours at 37°C with 5% CO_2_, and the media were collected and used immediately or stored frozen until use. In ARPE-19 cells, at the endpoint of the phagocytosis assay as described above, a total of 100 µL of the culturing medium was collected and used immediately or stored at –80°C until use. The levels of β-hydroxybutyrate (β-HB) released from the RPE cells were determined in the collected samples using the enzymatic activity of β-HB dehydrogenase in a colorimetric assay from the Beta-Hydroxybutyrate LiquiColor Assay (2440058; Stanbio Laboratory, Boerne, TX, USA) with β-HB standards and following the manufacturer's instructions.

### Free Fatty Acids Quantification Assay

A total of 50 µL of conditioned medium from ARPE-19 cell cultures was collected and used to quantify free fatty acids using the Free Fatty Acid Assay Kit–Quantification (ab65341; Abcam, Cambridge, MA, USA) following manufacturer's instructions.

### Statistical Analyses

Data were analyzed with the two-tailed unpaired Student's *t*-test or two-way ANOVA and are shown as mean ± SD. *P* < 0.05 was considered statistically significant.

## Results

### Generation of an RPE-Specific *Pnpla2*-KO Mouse

To circumvent the premature lethality of *PNPLA*2-KO mice,^32^ a mouse model with RPE-specific knockout of the *PNPLA*2 gene was designed. For this purpose, we crossed *Pnpla*2^f/f^ mice^23^ with *BEST1*-*cre* transgenic mice^24^ to obtain mice with conditional *Pnpla2* knockout specific to the RPE, hereafter referred to as cKO (or *Pnpla*2^f/f/Cre^). In the cKO mice, the promoter of the RPE-specific gene *VMD2* (human bestrophin, here referred to as *BEST1*) drives the expression of the Cre recombinase and restricts it to the RPE. These mice carry two floxed alleles in the *Pnpla*2 gene and a copy of the *BEST1*-*cre* transgene (*Pnpla*2^f/f/Cre^).

**Figure 1.**
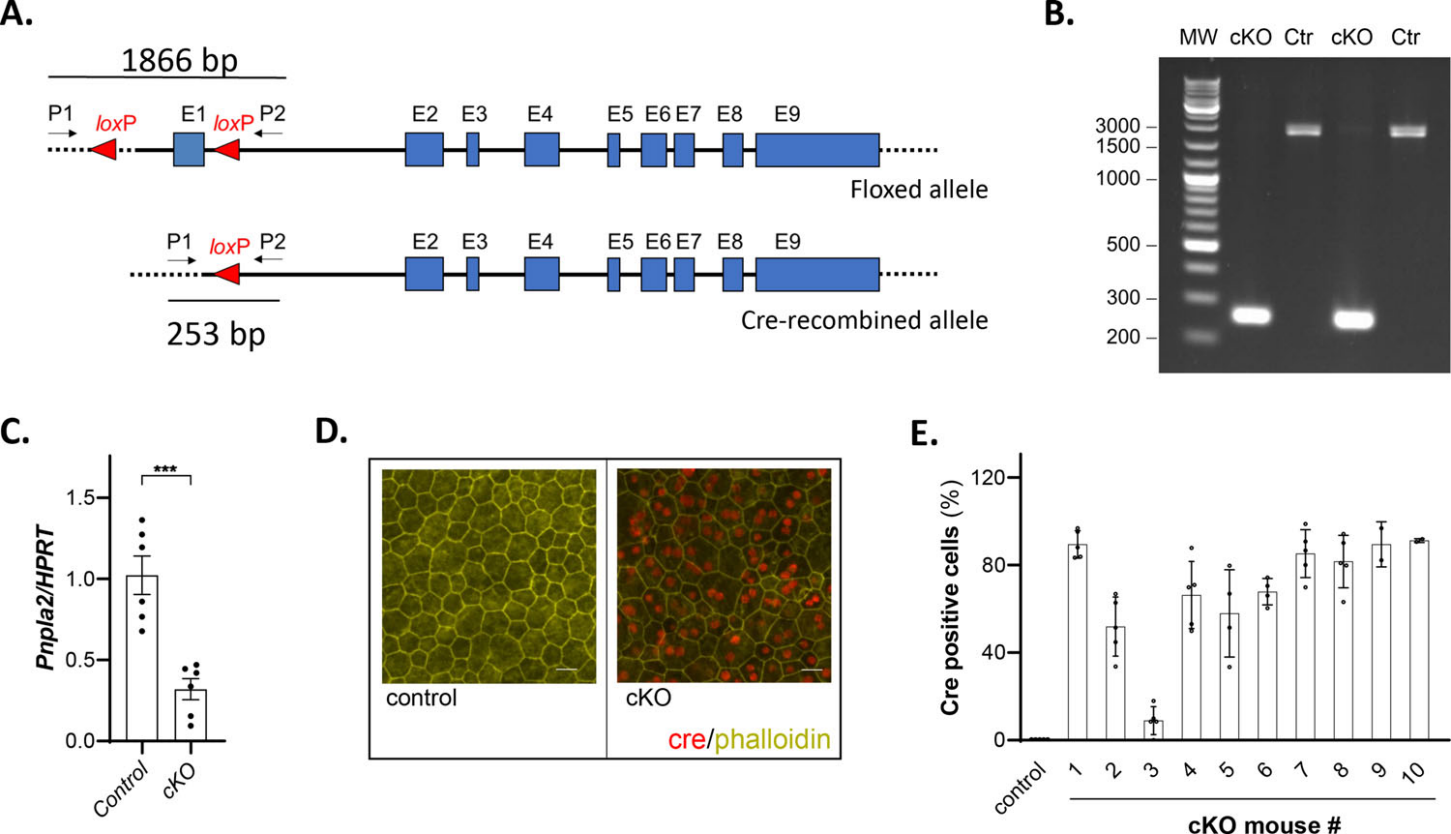
Generation of RPE-specific *PNPLA*2-cKO mice. (**A**) Scheme of *Pnpla*2 floxed and Cre-mediated recombined allele. The *loxP* sites flank exon 1. P1 and P2 are the primers homologous to sequences outside the floxed region (flanked by the *loxP* sites) used to detect Cre-mediated recombination (generating recombined alleles) on genomic DNA. The sizes of the amplicons obtained by PCR using P1 and P2 are indicated. (**B**) Gel electrophoresis of PCR reaction products obtained using primers P1 and P2 and genomic DNA isolated from mouse eyecups from either cKO or control (Ctr) mice (*Pnpla*2^f/+^); lane 1 (MW) corresponds to molecular weight markers (GeneRuler DNA Ladder Mix). One eyecup per lane from a 4-month-old mouse (*n* = 2 cKO, *n* = 2 Ctr). (**C**) *Pnpla*2 expression (vs. *HPRT*) in RPE from 1-month-old cKO mice (*Pnpla*2^f/f/Cre^) relative to control littermates (*Pnpla*2^f/f^). Each data point corresponds to the average of six PCR reactions per eyecup, six eyes from three cKO mice and six eyes from three control mice at 5 to 7 months old. (**D**) Cre (*red*) and phalloidin (*yellow*) labeling of RPE/choroid flatmounts from control (*Pnpla2*^f/f^) (*left*) and littermate cKO *(Pnpla2*^f/f/Cre^) (*right*) mice (*n* = 2 images from individual mouse eyecup at 11–14 months old). *Scale* *bar*: 20 µm. **(E)** Plot of percentage of Cre-positive RPE cells in cKO animals (*Pnpla2*^f/f/Cre^*^;^* *n* = 10; age, 10.5–18.5 months old) as indicated in the *x*-axis. Each data point corresponds to percentage of Cre-positive RPE cells from an ROI, each bar corresponds to a flatmount of an individual cKO mouse, and the bar for control (*Pnpla2*^f/f^) has data from 10 mice.

We performed PCR reactions with primers P1 and P2 upstream and downstream from the *loxP* sites flanking exon 1, respectively (Fig. 1A), with DNA extracted from cKO eyecups and found that the amplimers had the expected length of 253 bp corresponding to the recombined (cKO) allele (Fig. 1B), thus showing that the Cre–*loxP* recombination occurred successfully and led to deletion of the floxed region (exon 1) in the RPE of cKO mice (or *Pnpla*2^f/f/Cre^). Conversely, we observed two PCR bands of 1749 bp and 1866 bp for littermate *Pnpla*2^f/+^ control mice carrying a wild-type (WT) and a floxed allele, respectively (the floxed allele contained two *loxP* sites) (Fig 1B). In lanes for the cKO (or *Pnpla*2^f/f/Cre^), we also observed very low-intensity bands migrating at positions corresponding to 1749 bp and 1866 bp, which probably resulted from a few unsuccessful recombination events.

RT-PCR revealed *PNPLA2* transcript levels in the RPE that were lower from cKO mice than from control (with a mean that was about 32% of the control mice) (Fig. 1C). We determined the percentage of RPE cells that produced the Cre protein by immunofluorescence of RPE whole flatmounts. Cells were visualized by co-staining with fluorescein-labeled phalloidin antibody to detect the actin cytoskeleton. We observed Cre immunoreactivity in the RPE flatmounts isolated from cKO mice, whereas no Cre labeling was detected in the controls (Fig. 1D). The overall distribution was patchy and mosaic, as previously described for the *BEST1-cre* mice.^24^ The percentage of Cre-positive cells in the ROIs of the flatmounts showed nine mice with expected percentages of Cre-positive cells in the RPE and one with low Cre positivity (Fig. 1E). The average of the mean values of Cre-positive cells for each cKO mouse (mouse numbers 1, 2, 4–10) was 75% (range, 52%–91%), which was within the expected range for Cre positivity in the RPE of the *BEST1-cre* mouse.^24^ Cre-positive cells were not detected in the RPE of control animals (Figs. 1D, 1E). Unfortunately, further protein analysis of PEDF-R in mouse retinas was not conclusive because several commercial antibodies to PEDF-R gave high background by immunofluorescence and in western blots. Nevertheless, the results demonstrate the successful generation of RPE-specific *PNPLA*2 knockdown mice.

### Lipid Accumulates in the RPE of *Pnpla2*-cKO Mice

**Figure 2.**
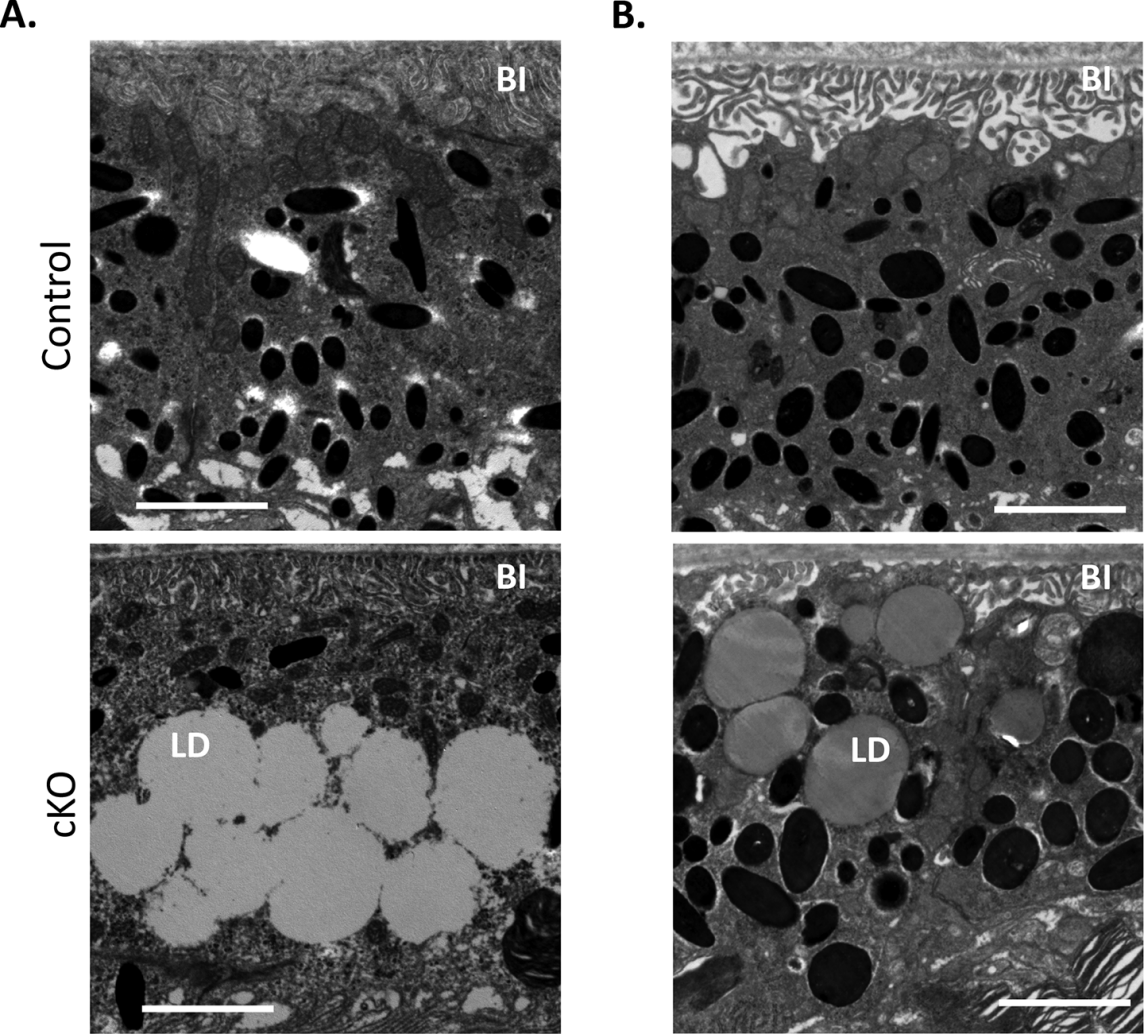
Lipid accumulation in the RPE of *Pnpla2*-cKO mice. Electron microscopy micrographs showing the RPE structure of 3-month-old (**A**) and 13-month-old (**B**) cKO mice and control animals. *Scale bar*: 2 µm. The representative images were selected among examinations of micrographs from eight eyes of cKO mice (*PNPLA2*^f/f/Cre+^), from seven eyes of *PNPLA2*^f/f^ control mice at 1.75 to 3.75 months old, and from three eyes of cKO mice and three eyes of control mice at 12.5 to 13 months old. LD, lipid droplets; BI, basal infoldings.

We examined the ultrastructure of the RPE by transmission electron microscopy (TEM) imaging. Accumulation of large lipid droplets (LDs) was observed in cKO mice as early as 3 months of age compared to the control mice cohort (Fig. 2A), and LDs were still observed in the RPE of 13-month-old *Pnpla*2-cKO mice compared to controls (Fig. 2B). The presence of LDs was associated with a lack of (normally seen in the basal side; see Supplementary Figs. S2A, S2H) or decreased thickness of the basal infoldings, and with granular cytoplasm, abnormal mitochondria (Supplementary Fig. S2B), and disorganized localization of organelles (mitochondria and melanosomes) (Supplementary Fig. S2A). In some cells, LDs crowded the cytoplasm and clustered the mitochondria and melanosomes into the apical region of the cells (Supplementary Figs. S2A, S2C, S2D); however, the number and expansion of LDs within the cells appeared to be random (Supplementary Fig. S2E). Normal apical cytoplasmic processes were lacking, and degeneration in the outer segment (OS) tips of the photoreceptors was apparent (Supplementary Figs. S2A, S2F). Additionally, normal phagocytosis of the OSs by RPE cells was not evident, implying a certain degree of impairment (Supplementary Figs. S2A, S2E, S2G). There were apparent unhealthy nuclei with pyknotic chromatin and leakage of extranuclear DNA, indicating the beginning of a necrotic process (Supplementary Fig. S2B). Some RPE cells had lighter low-density cytoplasm, indicating degeneration of cytoplasmic components in contrast to the denser and fuller cytoplasm in the RPE of the littermate controls (Supplementary Figs. S2I, S2J). Thus, these observations imply that *Pnpla2* downregulation caused lipid accumulation in the RPE.

### *Pnpla2* Deficiency Increases Rhodopsin Levels in the RPE of Mice

**Figure 3.**
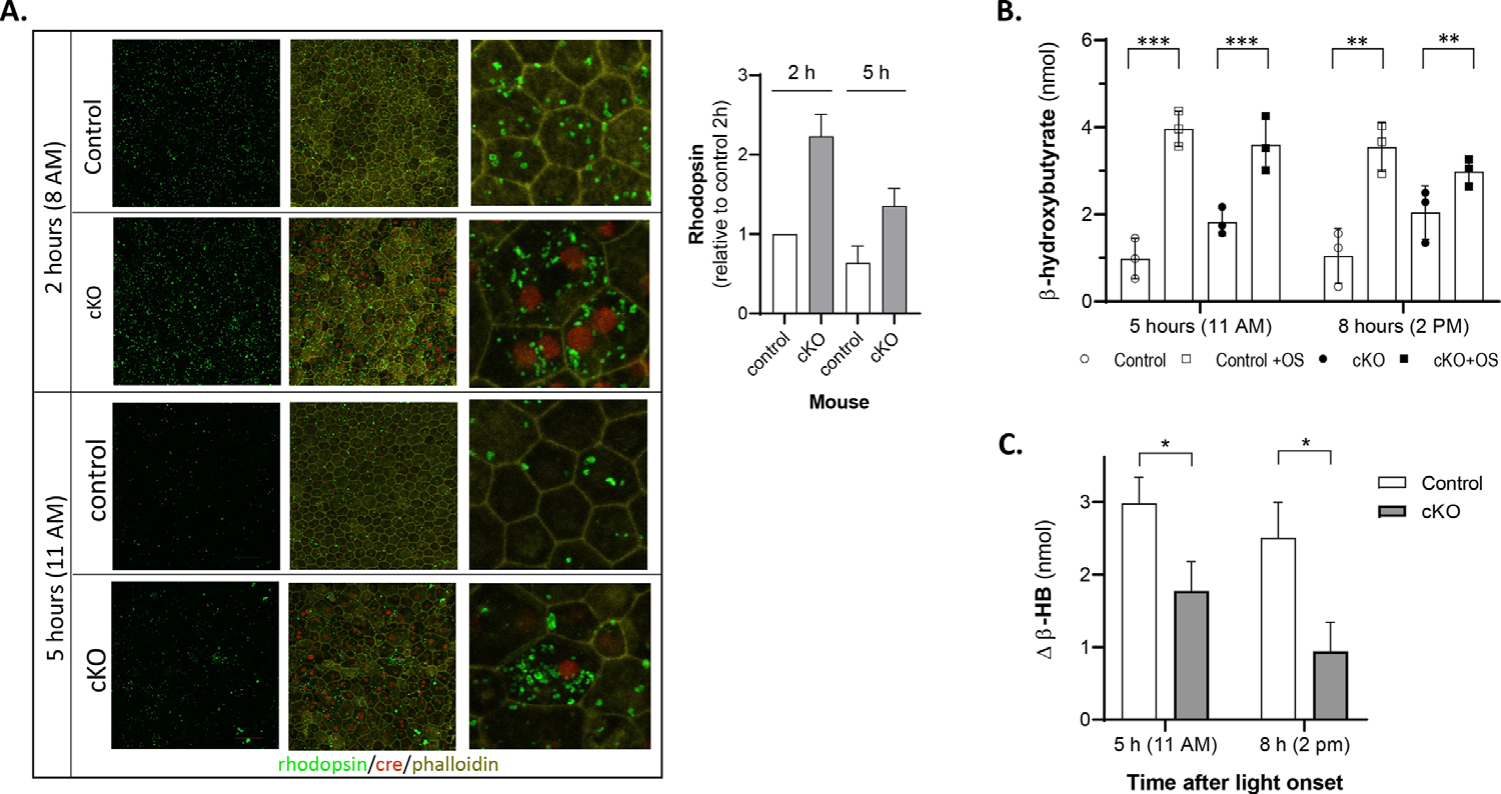
Phagocytosis and β-hydroxybutyrate production in the RPE of *Pnpla2*-cKO mice. (**A**) Representative ROIs of the eyecup from one control and one cKO animal isolated at 2 hours (8 AM) and 5 hours (11 AM) after light onset (6 AM) after immunolabeling for rhodopsin (*green*), phalloidin (*yellow*), and Cre (*red*). The column to the right shows magnification of an area. The mean of rhodopsin immunolabel intensity in micrographs (*n* ≥ 6 ROIs) from flatmounts (as indicated in the *x*-axis) relative to control at 2 hours was determined among three mice per condition and is shown in the plot. Age of mice was 10.5 to 18.5 months. (**B**) Ex vivo β-HB release by the RPE of *Pnpla*2-cKO eyecups upon ingestion of OSs in comparison to that of controls. Eyecups were isolated at 5 hours (11 AM) and 8 hours (2 PM) after light onset (6 AM). Statistical significance was calculated using two-way ANOVA for the two groups (controls and cKO mice) with and without treatment (second variance) for each time after light onset. ^*^P = 0.02, ^**^P = 0.006, ^***^P = 0.0001; ns, not significant; *n* = 6 eyecups from three control (f/+) mice at 3.5 months; *n* = 4 eyecups from two control (f/f/Cre–) mice at 3.5 months; *n* = 10 eyecups from five mice (f/f/Cre+) at 2.75 to 3.5 months. (**C**) The OS-mediated increase in β-HB release above base levels of the cKO RPE/choroid explants was calculated from the data in panel **C** and plotted.

Because the RPE does not express the rhodopsin gene, the level of rhodopsin protein in the RPE cells is directly proportional to their phagocytic activity.^5^^,^^33^ To investigate how the knockdown of *Pnpla2* affects RPE phagocytic activity in mice, we compared the rhodopsin-labeled particles present in the eyecup of cKO mice and those of control mice at 2 hours and 5 hours post-light onset in vivo. The ROIs for the mutant mice were selected from areas rich in Cre-positive cells. Phalloidin-labeled flatmounts of control mice (*n* = 10) showed that the RPE cells had the typical cobblestone morphology, whereas nine out of 10 of the cKO mice had distorted cell morphology. Rhodopsin was detected in all ROIs, and the labeled particles were more intense and larger in size in the majority of cKO flatmounts compared to those in the control mice. Representative ROIs are shown in Figure 3A. The observations implied that *Pnpla2* knockdown in the RPE prevented rhodopsin degradation in vivo.

### Ketogenesis Upon RPE Phagocytosis in Explants from cKO Mice Is Impaired

Given that RPE phagocytosis is linked to ketogenesis,^8^ we also measured the levels of ketone body β-HB released by RPE/choroid explants of the cKO mice ex vivo and compared them with those of control littermates. The experiments were performed at 5 hours (11 AM) and 8 hours (2 PM) after light onset, both times of day when the amount of β-HB released due to endogenous phagocytosis is not expected to vary with time. A phagocytic challenge by exposure to exogenous bovine OS increased the amount of β-HB released by explants from both cKO and control littermates compared to the β-HB released under base conditions (without the addition of exogenous OS) (Fig. 3B). The OS-mediated increase in β-HB release above base levels of the cKO RPE/choroid explants (1.8 nmol at 11 AM, 0.9 nmol at 2 PM) was lower than that for the control explants (3 nmol at 11 AM and 2.5 nmol at 2 PM) (Fig. 3C). These observations reveal a deficiency in β-HB production by the RPE/choroid explants of cKO mice under phagocytic challenge ex vivo.

### Electroretinography of the cKO Mouse

**Figure 4.**
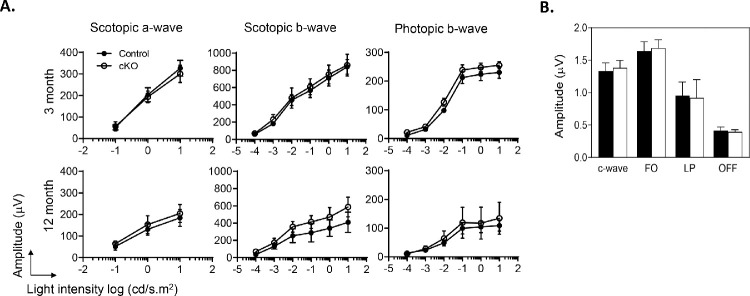
RPE and retinal functionality in RPE-*Pnpla2-*cKO mice. (**A**) ERG amplitude graphs of scotopic a- and b-waves and photopic b-waves, as a function of light intensity (*x*-axis) of 3- and 12-month-old cKO mice (*open circles*) and littermate controls (*Pnpla2*^f/f^, *closed circles*) (*n* = 3 per genotype). (**B**) Bar graph showing the amplitude (mean, SD) of the c-wave, fast oscillation (FO), light peak (LP), and off-response (OFF) measured by DC-ERG of 11-week-old cKO mice (*n* = 4, *open bars*) and *Pnpla2*^f/f^ and *Pnpla2*^f/+^ control mice (*n* = 5, *closed bars*).

To examine the functionality of the retina and RPE of cKO mice, we performed ERG and DC-ERG. Figure 4 shows histograms that revealed no differences among the animals, implying that the functionality was not affected in the RPE of *Pnpla*2-cKO mice.

### Phagocytic ARPE-19 Cells Engulf and Break Down POS Protein and Lipid

The complexity of the interactions that occur in the native retina makes it difficult to evaluate the subcellular and biochemical changes involved in phagocytosis of POSs. Cultured RPE cells provide an ideal alternative for performing these studies. Accordingly, we designed and validated an assay with a human RPE cell line, ARPE-19, to which we added POSs isolated from bovine retinas, as described in Methods. The lipid composition of the POSs fed to the ARPE-19 cells included phosphatidylcholine (PC) containing very-long-chain polyunsaturated fatty acids that was ∼27 relative mole percent of total PC species in the POSs. The other major PC species include PC 32:00, PC 40:06, and PC 54:10, comprising ∼38 relative mole percent of the total PC phospholipids. The most abundant phosphatidylethanolamine (PE) species in the POSs were PE 38:06, PE 40:05, and PE 40:06, which accounted for about 74 relative mole percent of the total PE phospholipids. The confluent monolayer of cells was exposed to the purified POS membranes for up to 2.5 hours, and then the ingested POSs were chased for 16 hours for pulse–chase experiments. The fate of rhodopsin, the main protein in POSs, was followed by western blotting of cell lysates. Rhodopsin was detected in the cell lysates as early as 30 minutes; its levels increased at 1 hour and 2.5 hours during the POS pulse and decreased with a 16-hour chase (Supplementary Fig. S3A). Quantification revealed that rhodopsin levels were 21% of those detected after 2.5 hours of POS supplementation (Supplementary Fig. S3B).

Free fatty acid and β-HB levels were also determined in the culture media during the pulse. The levels of free fatty acids in the medium of POS-challenged ARPE-19 cells were seven-, five-, and threefold higher after 30 minutes, 60 minutes, and 2.5 hours of incubation, respectively, relative to those in the medium of cells not exposed to POSs (Supplementary Fig. S3C). The β-HB levels released into the medium after POS addition also increased by 10-, 2.5-, and 4-fold after 30 minutes, 60 minutes, and 2.5 hours of incubation, respectively, relative to those observed in the medium of cells not exposed to POSs (Supplementary Fig. S3D). Altogether, these results show that, under the specified conditions in this study, the batch of ARPE-19 cells phagocytosed (i.e., engulfed and digested bovine POS protein and lipid components).

### Bromoenol Lactone Blocks the Degradation of POS Components in Phagocytic ARPE-19 Cells

**Figure 5.**
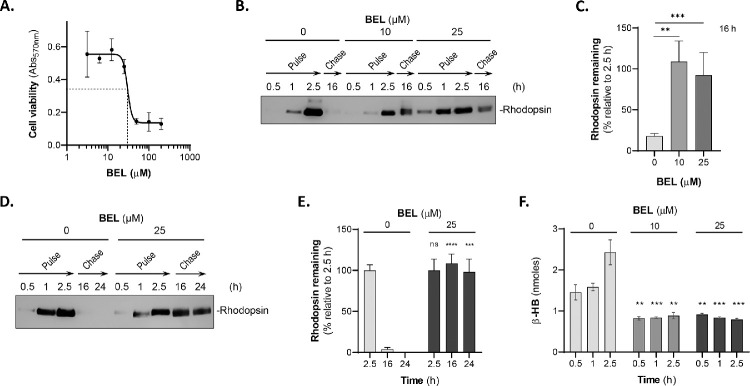
Phagocytosis in ARPE-19 cells pretreated with BEL. (**A**) ARPE-19 cells were incubated with BEL at the indicated concentrations for 3.5 hours. The mixture was then removed, washed gently with PBS, and incubated with complete medium for a total of 16 hours. Cell viability was assessed by crystal violet staining and with three replicates per condition. (**B**) Representative immunoblot of total lysates of cells pretreated with DMSO alone or 10- or 25-µM BEL/DMSO for 1 hour prior to pulse–chase of POSs, as described in Methods. Extracts of cells harvested at the indicated times (*top of blot*) were resolved by SDS-PAGE followed by immunoblotting with anti-rhodopsin. Migration position of rhodopsin is indicated to the *right of the blot*. (**C**) Quantification of rhodopsin from total lysates of cells of the pulse–chase experiments as in panel **B**. Samples from each biological replicate were resolved in duplicate by SDS-PAGE from two experiments and singly for the third experiment for quantification. Intensities of the immunoreactive bands were determined, and the percentages of the remaining rhodopsin after 16-hour chase relative to rhodopsin at 2.5-hour pulse were plotted. (**D**) Representative immunoblot of total lysates of cells, as in panel **B** to determine the effects of BEL at 16 hours and 24 hours of chase (as indicated). (**E**) Quantification of rhodopsin from two independent experiments of the pulse–chase experiments as in panel **D**. Samples from each biological replicate were resolved in duplicate by SDS-PAGE for quantification. Intensities of the immunoreactive bands were determined, and the percentages of the remaining rhodopsin after 16-hour chase relative to rhodopsin at 2.5-hour pulse were plotted. (**F**) Cells were preincubated with DMSO alone or 10- or 25-µM BEL/DMSO in Ringer's solution at 37°C for 1 hour. The mixture was then removed, and cells were incubated with Ringer's solution containing 5-mM glucose and POSs (1 × 10^7^ units/mL) with DMSO alone or 10- or 25-µM BEL/DMSO for the indicated times (*x*-axis). Media were removed to determine the levels of β-HB secretion, which are plotted on the *y*-axis. Data are presented as mean ± SD. ^**^P < 0.01, ^***^P < 0.001 (*n* = 3).

We investigated the role of PEDF-R PLA2 activity in RPE phagocytosis. As we have previously described, BEL, a calcium-independent phospholipase A2 inhibitor, inhibits PEDF-R PLA2 enzymatic activity.^15^ First, we determined the concentrations of BEL that would maintain viability of ARPE-19 cells. Figure 5A shows the concentration response curve of BEL on ARPE-19 cell viability. The BEL concentration range tested was between 3.125 µM and 200 µM, and the Hill plot estimated an IC_50_ (concentration that lowers cell viability by 50%) of 30.3 µM BEL. Therefore, to determine the effects of BEL on the ARPE-19 phagocytic activity, cultured cells were preincubated with the inhibitor at concentrations below the IC_50_ for cell viability prior to pulse–chase assays designed as described above. Pretreatment with DMSO alone without BEL was assayed as a control. Interestingly, the inhibitor at 10 µM and 25 µM blocked more than 90% of the degradation of rhodopsin during POS chase for 16 hours in ARPE-19 cells (Figs. 5B, 5C). Similar blocking effects of BEL (25 µM) were observed with time up to 24 hours during the chase (Figs. 5D, 5E). The inhibitor did not appear to affect rhodopsin ingestion. The rhodopsin levels in pulse–chase assays with cells pretreated with DMSO alone were like those without pretreatment (compare Fig. 5B and Supplementary Fig. S3A). The cells observed under the microscope after the chase point and prior to the preparation of cell lysates had similar morphology and density among cultures with and without POS and among cultures before and after pulse. Moreover, BEL blocked 40% of the β-HB releasing activity of ARPE-19 cells, whereas DMSO alone did not affect the activity (Fig. 5F). These observations demonstrate that, although binding and engulfment were not affected by BEL under the conditions tested, phospholipase A2 activity was required for rhodopsin degradation and β-HB release by ARPE-19 cells during phagocytosis.

### *PNPLA2* Downregulation in ARPE-19 Cells Impairs POS Degradation

**Figure 6.**
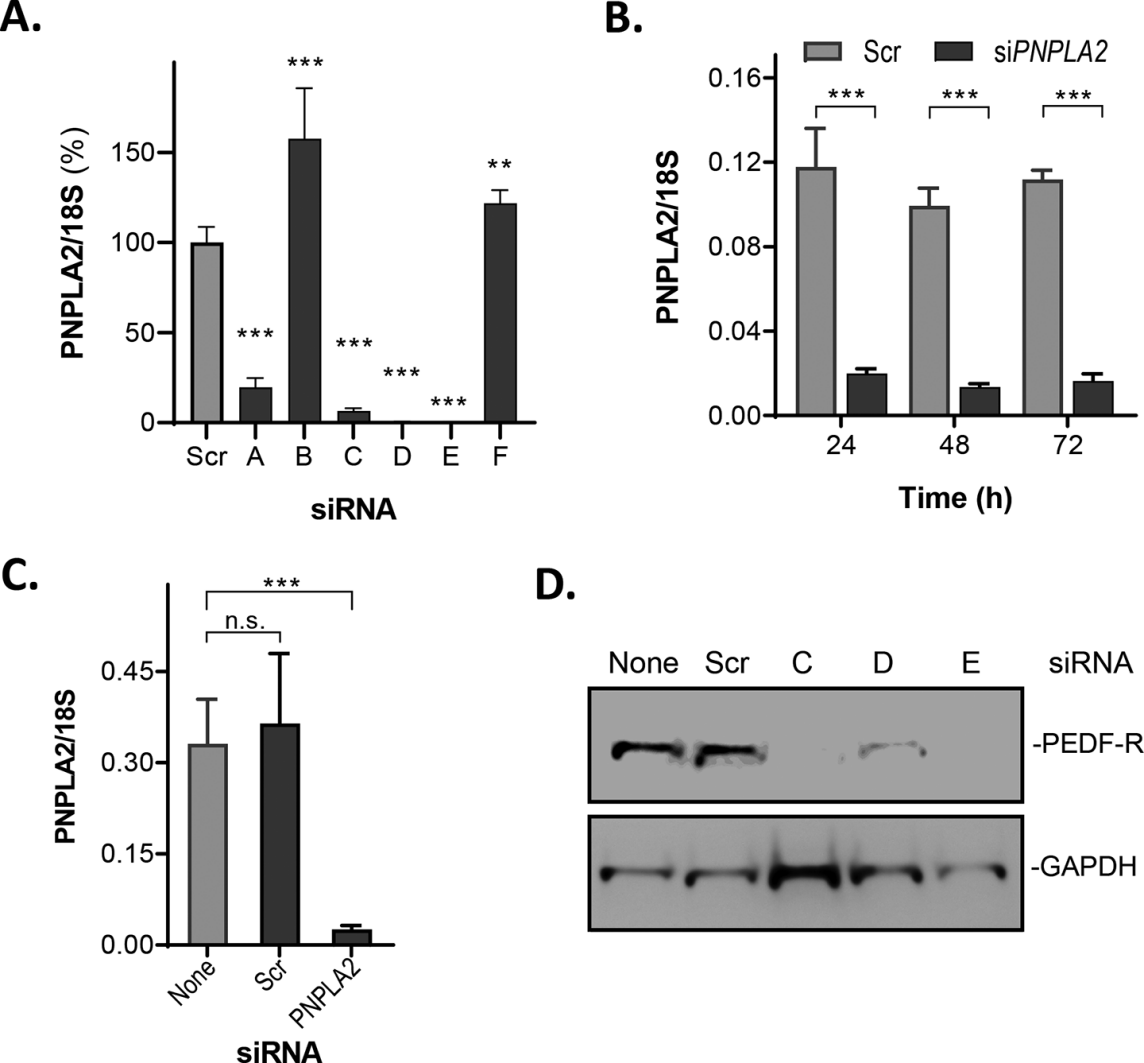
Knockdown of *PNPLA2* in ARPE-19 cells. ARPE-19 cells were transfected with Scr or siRNAs targeting *PNPLA2*, and mRNA levels and protein were tested. (**A**) RT-qPCR to measure *PNPLA2* mRNA levels in ARPE-19 cells 72 hours after transfection with Scr and six different siRNAs (as indicated on the *x*-axis) was performed, and a plot is shown. *PNPLA2* mRNA levels were normalized to 18S. All siRNA are represented as the percentage of the scrambled siRNA control (*n* = 3). (**B**) A plot is shown for a time course of *PNPLA2* mRNA levels following transfection with Scr and *siPNPLA2-C* (*n* = 3 (**C**) RT-qPCR of mock-transfected cells, cells transfected with Scr, and *siPNPLA2-C* (*x*-axis) at 72 hours after transfection. mRNA levels were normalized to the 18S RNA (*y*-axis) (*n* = 3). (**D**) Total protein was obtained from cells harvested 72 hours after transfection and resolved by SDS-PAGE followed by western blotting with anti-PNPLA2 and anti-GAPDH (loading control). The siRNAs used in the transfections are indicated at the top, and migration positions for PEDF-R and GAPDH are to the right of the blot. Data are presented as mean ± SD. ^**^P < 0.01, ^***^P < 0.001, ^***^P < 0.001.

We also silenced *PNPLA2* expression in ARPE-19 cells to investigate the possible requirement of PEDF-R for phagocytosis. First, we tested the silencing efficiency of six different siRNAs designed to target *PNPLA2*, along with a scramble siRNA sequence as negative control (see sequences in Table 3). The siRNA-mediated knockdown of *PNPLA2* resulted in significant decreases in the levels of *PNPLA2* transcripts (siRNA A, C, D, and E; Fig. 6A, Supplementary Fig. S5) with a concomitant decline in PEDF-R protein levels (siRNA C–E; Fig. 6D) in ARPE-19 cell extracts. The siRNAs with the highest efficiency for silencing *PNPLA2* mRNA (namely, C–E) were individually used for subsequent experiments and denoted as si*PNPLA2* (Fig. 6A). A time course of si*PNPLA2* transfection revealed that the gene was silenced as early as 24 hours and throughout 72 hours post-transfection and parallel to pulse–chase (98.5 hours) (Fig. 6B, Supplementary Fig. S5). There was no significant difference between mock transfected cells and cells transfected with Scr (Fig. 6C). Examining the cell morphology under the microscope, we did not notice differences between the scrambled and *siPNPLA2*-transfected cells. Western blots showed that protein levels of PEDF-R in ARPE-19 membrane extracts declined 72 hours after transfection (Fig. 6D). Thus, subsequent experiments with cells in which *PNPLA2* was silenced were performed 72 hours after transfection.

**Figure 7.**
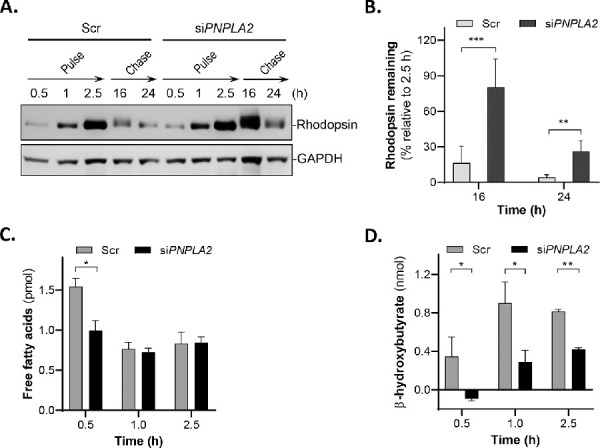
Phagocytosis and fatty acid metabolism in si*PNPLA2* cells. ARPE-19 cells were transfected with Scr or siRNAs targeting *PNPLA2.* At 72 hours after transfection, ARPE-19 cells were incubated with POSs (1 × 10^7^ units/mL) in 24-well tissue culture plates for pulse–chase experiments. (**A**) Representative immunoblot of total lysates of ARPE-19 cells at 0.5 hour, 1 hour, and 2.5 hours of POS pulse and at 16-hour and 24-hour chase periods, as indicated at the top of the blot. Proteins in cell lysates were subjected to immunoblotting with anti-rhodopsin followed by reprobing with anti-GAPDH as the loading control. (**B**) Quantification of rhodopsin from duplicate samples and three blots of cell lysates from pulse–chase experiments and time periods (indicated in the *x*-axis) as from panel. Data are presented as mean ± S.D; ^**^P < 0.01; ns, not significant. Intensities of the immunoreactive bands were determined, and the percentages of the remaining rhodopsin after 16-hour and 24-hour chase periods relative to rhodopsin at 2.5-hour pulse are plotted (*y*-axis). (**C**, **D**) Levels of secreted free fatty acids (**C**) and β-HB (**D**) were measured in culture media of cells transfected with Scr or *siPNPLA2* following incubation with POS for the indicated periods of times (*x*-axis). Data are presented as mean ± SD. ^*^P < 0.05, ^**^P < 0.01 (*n* = 3). Duplex si*PNPLA2* C was used to generate the data (see Table 3 for sequences of duplexes).

Second, we tested the effects of *PNPLA2* silencing on ARPE-19 cell phagocytosis. Here we monitored the outcome of rhodopsin in pulse–chase experiments. Interestingly, although *PNPLA2* knockdown did not affect ingestion, the si*PNPLA2*-transfected cells failed to degrade the ingested POS rhodopsin (88% and 24% remaining at 16 hours and at 24 hours, respectively), whereas Scr-transfected cells were more efficient in degrading them (21% and 12% remaining at 16 hours and 24 hours, respectively) (Figs. 7A, 7B).

Third, we also determined the levels of secreted free fatty acids and β-HB production in *PNPLA2* silenced cells at 0.5 hour, 1 hour, and 2.5 hours following POS addition. Free fatty acid levels in the culture medium were lower in *siPNPLA2*-transfected cells than in cells transfected with Scr 30 minutes after the addition of POSs, and no differences were observed between *siPNPLA2* and Scr at 1 hour and 2.5 hours after the addition of POSs (Fig. 7C). Secreted β-HB levels in the culture medium were lower in si*PNPLA2* cells than in Scr-transfected cells at all time points (Fig. 7D). To determine the effect of *PNPLA2* knockdown on lipid and fatty acid levels in the ARPE-19 cells fed POS membranes, we used ESI-MSMS and gas chromatography–flame ion detection to identify and quantify the total lipids and fatty acid composition of the ARPE-19 cells at 2.5 hours and 16 hours after POS feeding. Our results did not show any significant differences in the intracellular lipid and fatty acid levels in the *siPNPLA2* knockdown in Scr and WT control cells at either 2.5 hours or 16 hours after POS addition (data not shown). Taken together, these results demonstrate that digestion of POS protein and lipid components was impaired in *PNPLA2*-silenced ARPE-19 cells undergoing phagocytosis.

## Discussion

Here, we report that PEDF-R is required for efficient degradation of POSs by RPE cells after engulfment during phagocytosis. This conclusion is supported by the observed decrease in rhodopsin degradation, in fatty acid release, and in β-HB production upon POS challenge when the *PNPLA2* gene is downregulated or the PEDF-R lipase is inhibited. These observations occur in RPE cells in vivo, ex vivo, and in vitro. The findings imply that RPE phagocytosis depends on PEDF-R for the release of fatty acids from POS phospholipids to facilitate POS protein hydrolysis, thus identifying a novel contribution of this enzyme in POS degradation and, in turn, in the regulation of photoreceptor cell renewal.

This is the first time that the *PNPLA2* gene has been studied in the context of RPE phagocytosis of POSs. Previously, we investigated its gene product, PEDF-R, as a phospholipase-linked cell membrane receptor for pigment epithelium-derived factor (PEDF), a retinoprotective factor encoded by the *SERPINF1* gene and produced by RPE cells.^15^^,^^17^^,^^34^^,^^35^ Like RPE cells, non-inflammatory macrophages are phagocytic cells; however, unlike RPE cells, they are found in all tissues, where they engulf and digest cellular debris, foreign substances, bacteria, and other microbes, for example.^36^^,^^37^ The Kratky laboratory reported data on the effects of *PNPLA2* silencing in efferocytosis obtained using *PNPLA2*-deficient mice (*atgl*^−/−^ mice) and demonstrated that their macrophages have lower triglyceride hydrolase activity, higher triglyceride content, lipid droplet accumulation, and impaired phagocytosis of bacterial and yeast particles,^21^ and that in these cells intracellular lipid accumulation triggers apoptotic responses and mitochondrial dysfunction.^38^ We have shown that *PNPLA2* gene knockdown causes RPE cells to be more responsive to oxidative stress-induced death.^39^ *PNPLA2* gene silencing, PEDF-R peptides blocking ligand binding, and enzyme inhibitors abolish the activation of mitochondrial survival pathways by PEDF in photoreceptors and other retinal cells.^17^^,^^34^^,^^40^ Consistently, overexpression of the *PNPLA2* gene or exogenous additions of a PEDF-R peptide decreases both the death of RPE cells undergoing oxidative stress and the accumulation of biologically detrimental leukotriene LTB4 levels.^31^ The fact that PEDF is a ligand that enhances PEDF-R enzymatic activity suggests that exposure of RPE to this factor is likely to enhance phagocytosis. These implications are unknown and require further study. Exogenous additions of recombinant PEDF protein to ARPE-19 cells undergoing phagocytosis did not provide evidence for such enhancement (JB, personal observations). This suggests that heterologous *SERPINF1* overexpression in cells and/or an animal model of inducible knock-in of *Serpinf1* may be useful to focus on the role of PEDF/PEDF-R in RPE phagocytosis unbiased by the endogenous presence of PEDF.

To investigate the consequences of *PNPLA2* silencing in POS phagocytosis, we generated a mouse model with a targeted deletion of *Pnpla2* in RPE cells in combination with the *BEST-cre* system for its exclusive conditional silencing in RPE cells (cKO mouse). These mice are viable with no apparent changes in other organs or in weight compared with control littermates and WT mice. The cKO mice live to an advanced age, in contrast to the constitutively silenced *PNPLA2*-KO mice in which the lack of the gene causes premature lethality (12–16 weeks) due to heart failure associated with massive accumulation of lipids in cardiomyocytes.^32^ The RPE cells of the cKO mouse have large lipid droplets at early and late ages (Fig. 2A, Supplementary Fig. S2) consistent with a buildup of substrates for the lipase activities of the missing enzyme. In cKO mice, lipid accumulation is associated with a lack of or decreased thickness of the basal infoldings, granular cytoplasm, abnormal mitochondria, and disorganized localization of organelles (mitochondria and melanosomes) in some RPE cells (Supplementary Fig. S2). Taken together, the TEM observations in combination with the greater rhodopsin accumulation and decline in β-HB release in cKO mice support the notion that PEDF-R is required for lipid metabolism and phagocytosis in the RPE. However, interestingly, the observed features do not seem to affect photoreceptor functionality (Supplementary Fig. S3) and appear to be inconsequential to age-related retinopathies in the *Pnpla2*-cKO mouse. This unanticipated observation suggests that the remaining RPE cells expressing the *Pnpla2* gene probably complement activities of those lacking the gene, thereby lessening photoreceptor degeneration and dysfunction in the cKO mouse. We note that the cKO mouse has a mosaic expression pattern with non-Cre-expressing RPE cells, as shown before for the *BEST1-cre* transgenic line.^24^ At the same time, the ERG measurements performed correspond to global responses of the photoreceptors and RPE cells, thereby missing individual cell evaluation. The lack of photoreceptor dysfunction with RPE lipid accumulation due to *PNPLA2* downregulation also suggests that during development a compensatory mechanism independent of *Pnpla2*/PEDF-R is likely to be activated, thereby minimizing retinal degeneration in the cKO mouse. Further study will be required to understand the implications of these unexpected findings. Animal models of constitutive heterozygous knockout or inducible knockdown of *PNPLA2* may be instrumental in addressing the role of *PNPLA2*/PEDF-R in mature photoreceptors unbiased by compensatory mechanisms due to low silencing efficiency or during development.

Results obtained from experiments using RPE cell cultures further establish that PEDF-R deficiency affects phagocytosis. It is worth mentioning that the data obtained under our experimental conditions were essentially identical to those typically obtained in assays performed with cells attached to porous permeable membranes, and this provides an additional advantage to the field by requiring shorter time to complete (see Supplementary Fig. S4). On one hand, the decrease in the levels of β-HB and in the release of fatty acids (the breakdown products of phospholipids and triglycerides) upon POS ingestion by cells pretreated with BEL, as well as those transfected with si*PNPLA2*, relative to the control cells indicates that *PNPLA2* participates in RPE lipid metabolism. On the other hand, the fact that PEDF-R inhibition and *PNPLA2* downregulation impair rhodopsin breakdown from ingested POS in RPE cells implies a likely dependence of PEDF-R-mediated phospholipid hydrolysis for POS protein proteolysis. In this regard, we envision that proteins in POS are mainly resistant to proteolytic hydrolysis, because the surrounded phospholipids block their access to proteases for cleavage. Phospholipase A2 activity would hydrolyze these phospholipids to likely liberate the proteins from the phospholipid membranes so they become available to proteases, such as cathepsin D, an aspartic protease responsible for 80% of rhodopsin degradation.^41^ It is important to note that the findings cannot discern whether PEDF-R is directly associated with the molecular pathway of rhodopsin degradation or indirectly involved in downregulating cathepsin D or other proteases. It is also possible that *PNPLA2* deficiency results in the alteration of critical genes regulating the phagocytosis pathway, such as light chain 3 (LC3) and genes of the mTOR pathway. Animal models deficient in such genes display retinal phenotypes such as impaired phagocytosis and lipid accumulation, similar to those observed in PEDF-R deficient cells.^42^^–^^44^ These implications require further exploration.

Given that BEL is an irreversible inhibitor of iPLA2, it has been used to discern the involvement of iPLA2 in biological processes. Previously, we demonstrated that BEL at 1 to 25 µM blocks 20% to 40% of the PLA activity of human recombinant PEDF-R.^15^ Jenkins et al.^18^ showed that 2-µM BEL inhibits >90% of the triolein lipase activity of human recombinant PEDF-R (referred to by this group as iPLA2ζ). In cell-based assays, Wagner et al.^45^ showed that BEL at 20 µM inhibits 40% of this enzyme's triglyceride lipase activity in hepatic cells. In the present study, to minimize cytotoxicity and ensure inhibition of the iPLA2 activity of PEDF-R in ARPE-19 cells, we selected BEL concentrations of 10 µM and 25 µM, which are below the IC_50_ determined for ARPE-19 cell viability (30.2 µM BEL) (Fig. 5A). We note that these BEL concentrations are within the range used in an earlier study on ARPE-19 cell phagocytosis.^22^ We compared our results to those by Kolko et al.^22^ regarding BEL effects on the phagocytosis of ARPE-19 cells. Using Alexa-red-labeled POSs, they reported that the percent of phagocytosis inhibition caused by 5- to 20-µM BEL was 24% in ARPE-19 cells. However, the authors did not specify the time of incubation for this experiment, and, based on the other experiments in the report, the time period may have included at least 12 hours of pulse, implying inhibition of ingestion of POSs. Also, there was no description of the effects of BEL on POS degradation. With unmodified POSs in pulse–chase assays, our findings showed a percent of inhibition after chase of >90% for 10-µM and 25-µM BEL, indicating more effective inhibition of POS digestion. The effect of BEL on POS ingestion under 2.5 hours was insignificant and over 2.5 hours remains unknown (pulse).

In addition, we show that pretreatment with BEL results in a decrease in the release of β-HB, which is produced from the oxidation of fatty acids liberated from POS. Thus, our assay adds new information, including pulse–chase, use of unmodified POSs, and β-HB release, to that reported by Kolko et al.^22^ It can be concluded that BEL can impair phagocytic processes in ARPE-19 cells. Although BEL is recognized as a potent inhibitor of iPLA2, it can also inhibit non-PLA2 enzymes, such as magnesium-dependent phosphatidate phosphohydrolase and chymotrypsin.^46^^,^^47^ Consequently, a complementary genetic approach targeting PEDF-R is deemed reasonable and appropriate to investigate its role in RPE phagocytosis. The complex and highly regulated phagocytic function of the RPE also serves to protect the retina against lipotoxicity. By engulfing lipid-rich POSs and using ingested fatty acids for energy, the RPE prevents the accumulation of lipids in the retina, particularly phospholipids, which could trigger cytotoxicity when peroxidized.^48^^,^^49^ In this regard, the lack of observed differences in intracellular phospholipid and fatty acids between PEDF-R-deficient RPE and control cells lead us to speculate that in ARPE-19 cells exposed to POS the undigested lipids remain within the cells and contribute to the total lipid and fatty acid pool, some of which may be converted to other lipid byproducts to protect against lipotoxicity. Also, the duration of the in vitro chase is shorter than what pertains in vivo, where undigested POSs accumulate and overtime coalesce to form the large lipid droplets observed in the RPE in vivo. Thus, future experiments aimed at detailed time-dependent characterization of specific lipid species and free fatty acid levels in the RPE in vivo and in media and cells in vitro will provide a better understanding of classes of lipids and fatty acids that contribute to lipid droplet accumulation in the RPE in vivo due to *PNPLA2* deletion. Nonetheless, the role of PEDF-R in POS degradation agrees with the previously reported involvement of phospholipase A2 activity in the RPE phagocytosis of POSs^22^ and with the role of providing protection by photoreceptors against lipotoxicity.

In conclusion, this is the first study, to our knowledge, to identify a role for PEDF-R in RPE phagocytosis. The findings imply that efficient RPE phagocytosis of POS requires PEDF-R, thus highlighting a novel contribution of this protein in POS degradation and its consequences in the regulation of photoreceptor cell renewal.

## Supplementary Material

Supplement 1
