# Supplementary material for: Degradation of Photoreceptor Outer Segments by the Retinal Pigment Epithelium Requires Pigment Epithelium-Derived Factor Receptor (PEDF-R)
Source: Invest Ophthalmol Vis Sci. 2021 Feb 19;62(2):30. doi: 10.1167/iovs.62.2.30 (PMC7900850; doi:10.1167/iovs.62.2.30)
Supplement: Supplement 1 [file iovs-62-2-30_s001.pdf]

Figure S1. SDS-PAGE and western blot of bovine POS

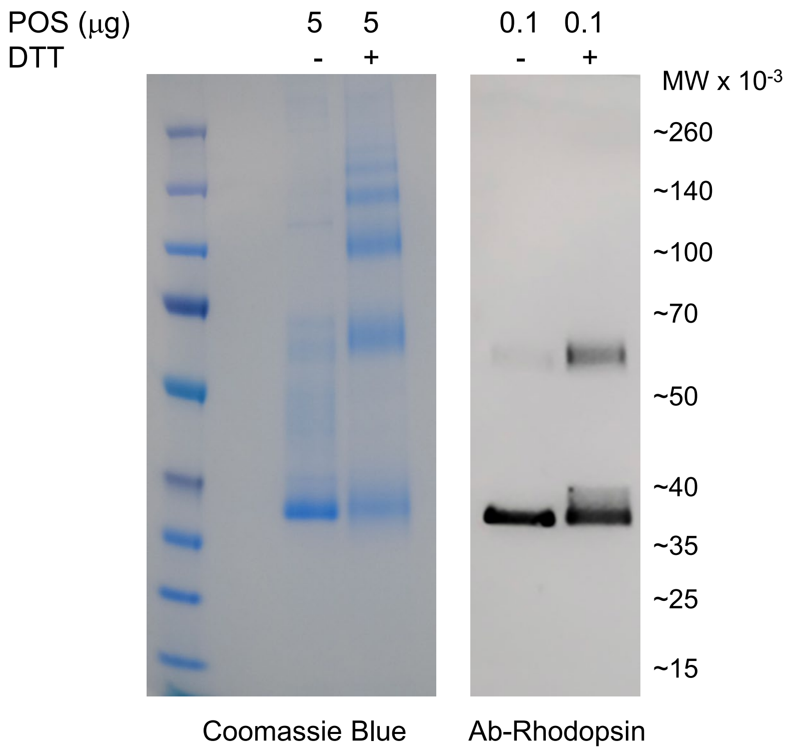

Proteins in the POS samples were determined and resolved by SDS-PAGE in the same gel in two sets: one with 5 μg and another with 0.1 μg protein per lane. For each set, one sample was non-reduced and the other was reduced with DTT. After electrophoresis, the gels were cut in half lengthwise. The gel portion with 5 μg of protein was stained with Coomassie Blue and the other portion with 0.1 μg protein was transferred to a nitrocellulose membrane for immunostaining using anti-rhodopsin antibodies (as described in Methods). Photos of the stained gel and western blot are shown.
The proteins of POS isolated from bovine retina had the expected migration pattern for both reduced and non-reduced conditions, and the main bands stained with Coomassie Blue comigrated with rhodopsin-immunoreactive proteins in western blots of POS proteins.

Figure S2. TEM of RPE in RPE-*Pnpla2*-cKO mice

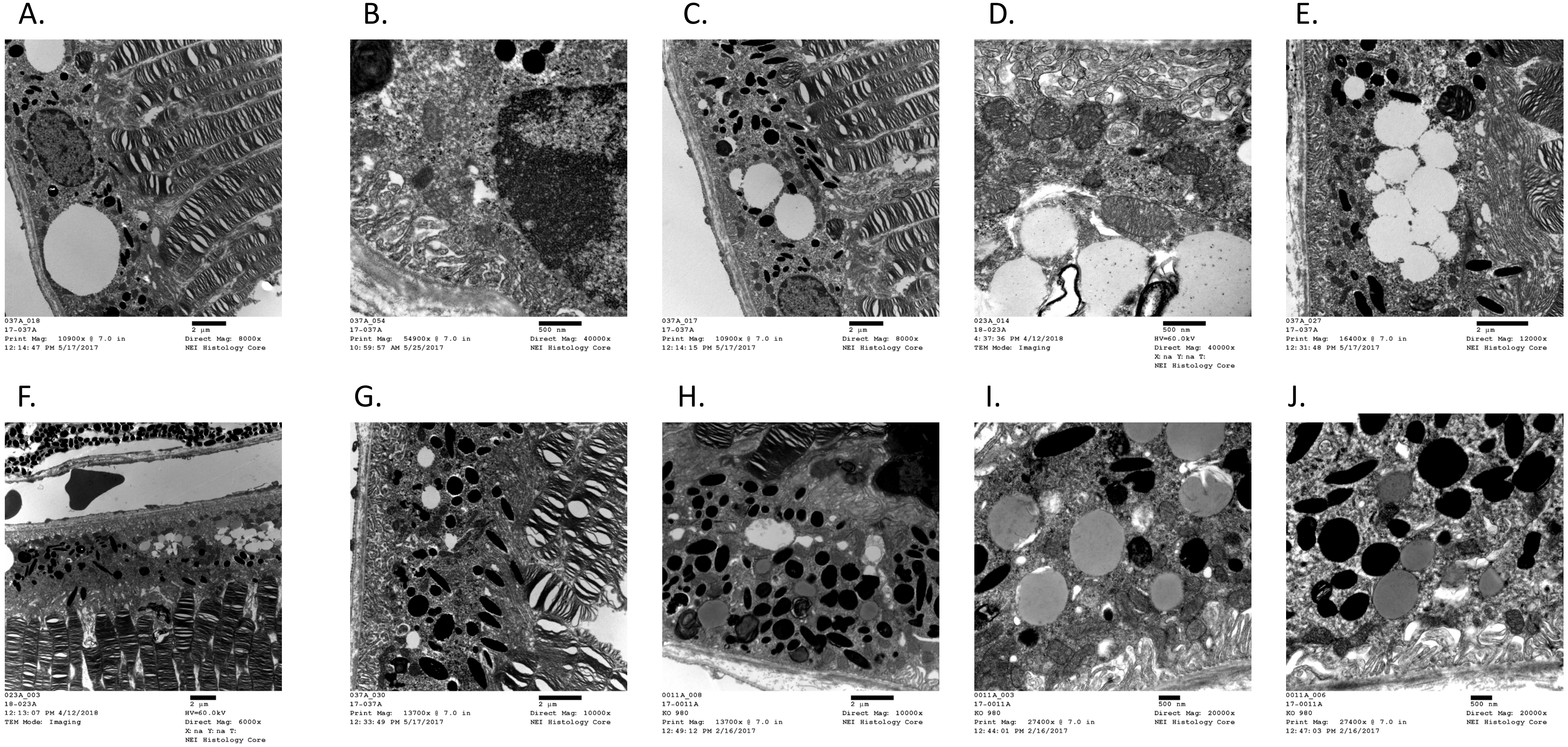

The presence of LDs was associated with lack (Fig. S2A) of or the decreased thickness of the basal infoldings, and with granular cytoplasm, abnormal mitochondria (Fig. S2B), and disorganized localization of organelles (mitochondria and melanosomes) (Fig. S1A). In some cells, the large LDs crowded the cytoplasm and clustered together the mitochondria and melanosomes into the apical region of the cells (Figs. S2A, S2C, S2D); however, LDs number and expansion within the cells appeared to be random and their expansion could go into any direction (Fig. S2E). Normal apical cytoplasmic processes were lacking; however, degeneration in the outer segment (OS) tips of the photoreceptors was visible (Figs. S2A, S2F); . Additionally, normal phagocytosis of the OS was lacking indicating an impaired RPE phagocytosis (Figs. S2A, S2E, S2G). There were apparent unhealthy nuclei with pyknotic chromatin and leakage of extranuclear DNA (enDNA), indicating that the beginning of the necrotic process had started (Fig. S2B). Some RPE cells lacked basal infoldings, normally seen at the basal side (Fig. S2H). Occasionally some RPE cells had lighter low-density cytoplasm indicating degeneration of cytoplasmic components in contrast to the denser and fuller cytoplasm in the RPE of the littermate control (Fig. S2I, S2J).

Figure S3.

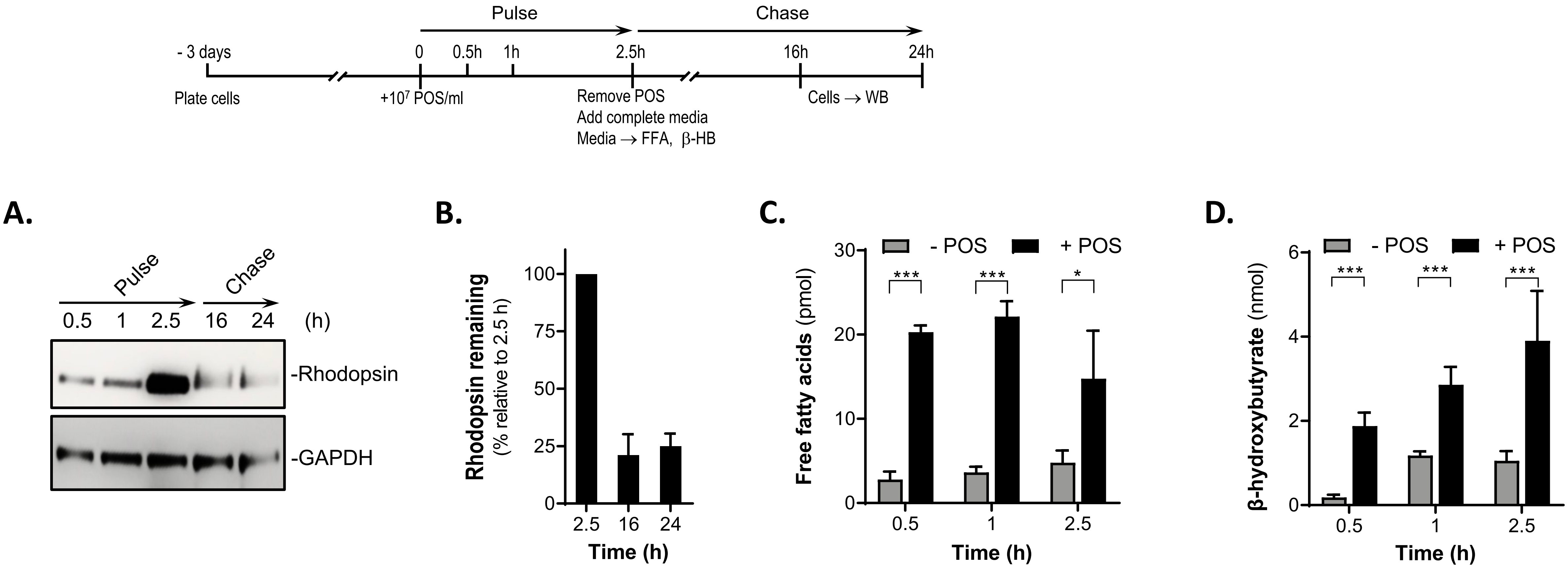

Figure S3.

Phagocytosis in ARPE-19 cells. ARPE-19 cells were cultured in 24-well plates for 3 days, and then exposed to POS at 1x107 units/ml for up to a 2.5-h pulse followed by an upto 24-h chase period as described in Methods. (A) Representative immunoblots of total cell lysates during pulse-chase (times indicated at the top of the blot) with anti-rhodopsin followed by reprobing with anti-GAPDH as the loading control are shown. Migration positions of rhodopsin and GAPDH are indicated to the right of the blot. Duplicate biological replicates were performed. (B) Quantification of rhodopsin from duplicate samples per condition from pulse-chase experiments at time periods indicated in the x-axis as from panel (A). Intensities of the immunoreactive bands from duplicate samples of cell lysates were determined. The percentage of the remaining rhodopsin after 16-h chase relative to rhodopsin at 2.5 h-pulse was plotted. (C-D) Levels of free fatty acids (C) and [illegible]-HB (D) measured in culture media of cells incubated with and without POS for the indicated periods of time (x-axis) were plotted and shown. n = 3 Data are presented as means ± S.D. * $p < 0.05$, ***$p<0.001$.

Figure S4.

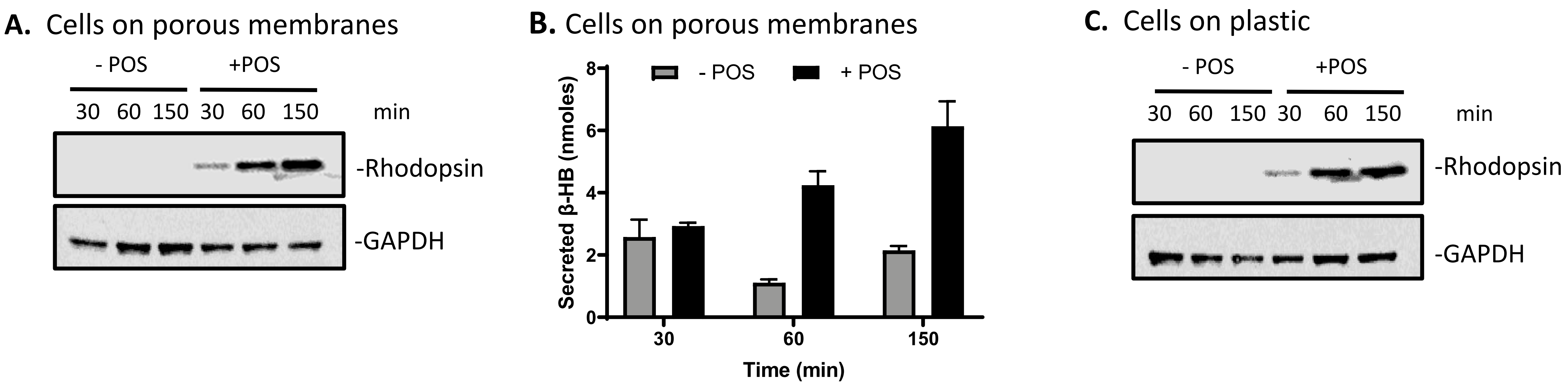

Figure S5. Phagocytosis in ARPE-19 cells in porous membranes. ARPE-19 cells were treated with $1x10^7$ POS/ml. (A) Representative immunoblot showing rhodopsin internalization from total cell lysates of ARPE-19 cells following 30, 60, and 150 min of POS incubation following plating in 12-well transwell inserts for 3 weeks. Cell extracts were resolved by SDS-PAGE followed by immunoblotting with anti-rhodopsin. The blot was stripped and reprobed with anti-GAPDH as a loading control. (B) Levels of B-HB secreted towards the apical membrane of ARPE-19 cells following POS incubation for 30, 60, and 150 min. Data are presented as means ± S.D.

ARPE-19 cells plated on porous membranes engulf bovine outer segments

To demonstrate a functional assay to study phagocytosis in ARPE-19 cells we perform the assay with confluent cells attached on porous membranes

Methods:

ARPE-19 cells seeded on porous membranes were incubated for 3 weeks in culturing media. Then the media was replaced with Ringer's solution alone or Ringer's solution containing 1×10^7 POS/ml and 5 mM glucose for the indicated time points. Rhodopsin was detected by western blotting.

Rhodopsin levels in the lysates of cells incubated with POS were detected in as little as 30 min and up to 2.5 h following POS incubation, while rhodopsin was undetectable in cells without POS (**Fig. S4A**). B-HB levels released into the media of the apical chamber of transwells following POS incubation increased four-fold and three-fold after 60 and 150 min, respectively, while released B-HB levels from cells incubated with Ringer's solution alone did not increase (**Fig. S4B**).

Figure S5.

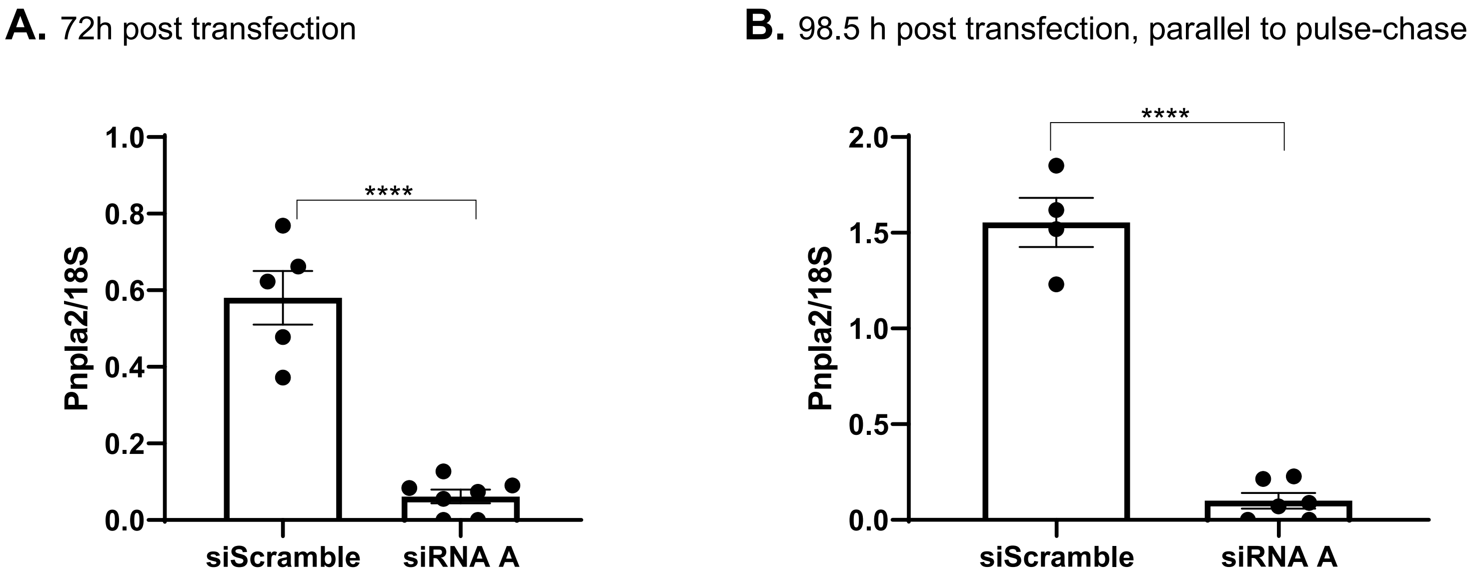

ARPE-19 cells were transfected with siScramble siRNA control or siRNAs targeting *PNPLA2 (siPNPLA2 A)*. RT-qPCR to measure *PNPLA2* mRNA levels in ARPE-19 cells at **(A)** 72 h post-transfection and (**B**) 98.5h post transfection equivalent to pulse (2.5h) and chase (24h) was performed with siRNA duplexes (as indicated in the *x*-axis). Treatment of cells in panel B was as for pulse-chase (see diagram in Fig S3). *PNPLA2* mRNA levels were normalized to 18S. n =3 biological replicates, each data point corresponds to the average of triplicate PCR reactions. The RT-PCR was repeated twice per biological replicate. Values that fell out of the standard curve were not included in the plot.

The data shows that si*PNPLA2* duplex silenced *PNPLA2* in ARPE-19 at 72 h post-transfection and that silencing was maintained throughout a 2.5 h and pulse-chase of 24 h.
